# Association between Metformin Use and Clinical Outcomes Following Pancreaticoduodenectomy in Patients with Type 2 Diabetes and Pancreatic Ductal Adenocarcinoma

**DOI:** 10.3390/jcm9061953

**Published:** 2020-06-22

**Authors:** Daegwang Yoo, Nayoung Kim, Dae Wook Hwang, Ki Byung Song, Jae Hoon Lee, Woohyung Lee, Jaewoo Kwon, Yejong Park, Sarang Hong, Jong Woo Lee, Kyungyeon Hwang, Dakyum Shin, Eunyoung Tak, Song Cheol Kim

**Affiliations:** 1 Division of Hepatobiliary and Pancreatic Surgery, Department of Surgery, Asan Medical Center, University of Ulsan College of Medicine, Seoul 05505, Korea; yoodaegwang@naver.com (D.Y.); drdwhwang@gmail.com (D.W.H.); mtsong21c@naver.com (K.B.S.); gooddr23@naver.com (J.H.L.); ywhnet@gmail.com (W.L.); skunlvup@naver.com (J.K.); blackpig856@gmail.com (Y.P.); 8thofnovember@hanmail.net (S.H.); hy_thename@hanmail.net (J.W.L.); hkyz84@gmail.com (K.H.); gracedkshin@gmail.com (D.S.); 2 Department of Clinical Epidemiology and Biostatistics, Asan Medical Center, University of Ulsan College of Medicine, Seoul 05505, Korea; nyny0803@amc.seoul.kr; 3 Department of Convergence Medicine, Asan Medical Institute of Convergence Science and Technology (AMIST), Asan Medical Center, University of Ulsan College of Medicine, Seoul 05505, Korea; 4 Division of Hepatobiliary and Pancreatic Surgery, Department of Surgery, Asan Medical Institute of Convergence Science and Technology (AMIST), Asan Medical Center, University of Ulsan College of Medicine, Seoul 05505, Korea

**Keywords:** metformin, pancreaticoduodenectomy, type 2 diabetes, pancreatic cancer

## Abstract

Retrospective studies on the association between metformin and clinical outcomes have mainly been performed on patients with non-resectable pancreatic ductal adenocarcinoma and may have been affected by time-related bias. To avoid this bias, recent studies have used time-varying analysis; however, they have only considered the start date of metformin use and not the stop date. We studied 283 patients with type 2 diabetes and pancreatic ductal adenocarcinoma following pancreaticoduodenectomy, and performed analysis using a Cox model with time-varying covariates, while considering both start and stop dates of metformin use. When start and stop dates were not considered, the metformin group showed significantly better survival. Compared with previous studies, adjusted analysis based on Cox models with time-varying covariates only considering the start date of postoperative metformin use showed no significant differences in survival. However, although adjusted analysis considering both start and stop dates showed no significant difference in recurrence-free survival, the overall survival was significantly better in the metformin group (Hazard ratio (HR), 0.747; 95% confidence interval (CI), 0.562–0.993; *p* = 0.045). Time-varying analysis incorporating both start and stop dates thus revealed that metformin use is associated with a higher overall survival following pancreaticoduodenectomy in patients with type 2 diabetes and pancreatic ductal adenocarcinoma.

## 1. Introduction

Metformin is an oral hypoglycemic drug widely used in patients with type 2 diabetes due to its affordability and efficacy. As metformin has been used for a long time, physicians have gained extensive experience and data regarding its efficacy and side effects. With respect to pancreatic cancer, metformin has been found to prevent cancer growth by inhibiting fibrosis and inflammation in several laboratory studies [1,2,3,4,5,6,7]. Specifically, metformin inhibits fibrosis by reducing TGF-β signaling in pancreatic stem cells and reduces inflammation by activating the AMPK pathway and inhibiting the mTOR pathway [1,3]. Given its proven safety profiles, if metformin is demonstrated to have positive effects on the oncological outcomes of pancreatic cancer, it may be readily used in clinical practice.

Studies have indicated that metformin use may be associated with improved clinical outcomes in various types of cancers [8,9,10,11,12,13,14]. Moreover, several randomized controlled studies (RCTs) [15,16,17], as well as systematic reviews and meta-analyses [18,19,20,21,22,23,24], have indicated the beneficial effects of metformin on the clinical outcomes of pancreatic cancer patients. However, the results of these studies have been found to be somewhat inconsistent, and there are certain issues relating to time-related bias in retrospective studies on metformin use [25]. Although three prospective trials on metformin use have been unaffected by time-related bias, these trials have only evaluated the effects of metformin in patients with unresectable pancreatic cancer. Moreover, two of these trials [15,26] included both diabetic and non-diabetic patients, whereas the third [17] was an uncontrolled trial and did not present the data for hazard ratios. Only a relatively few studies have investigated the effect of metformin use after curative resection in patients with resectable pancreatic cancer using limited time-varying analysis and in homogeneous populations of patients with left-sided (tail or body) and right-sided (head) pancreatic cancer. Although Chaiteerakij et al. have claimed that analysis based on Cox models with time-varying covariates may be able to solve the problem of time-related bias, they only considered the start date of metformin use and not the stop date [27]. Furthermore, the “ever versus never” classification of drug usage may lead to time-related bias, such as survival bias and immortal time bias, thereby leading to a possible overestimation of the survival benefit of metformin use [27,28], although patients in “ever” groups can survive for a sufficient length of time to receive metformin and gain a survival advantage [27]. In addition, conventional Cox analysis cannot be used to compare survival benefit based on the duration of metformin treatment [25,27]. Based on the comprehensive review of the studies on metformin and pancreatic ductal adenocarcinoma (PDAC), we performed analyses using Cox models with time-varying covariates that incorporate both the start and stop dates of metformin use.

In this study, we aimed to determine the effect of metformin on the survival of patients with type 2 diabetes who underwent pancreaticoduodenectomy (PD) due to PDAC in the pancreatic head, and attempted to avoid time-related bias in analysis by considering both the start and stop dates of metformin use.

## 2. Methods

### 2.1. Patient Database

We retrospectively reviewed the data of all preoperatively diagnosed patients with type 2 diabetes who underwent PD due to PDAC in the pancreatic head between May 2007 and July 2016 at the Asan Medical Center (Seoul, Korea). For the purposes of analysis, we collected data relating to patient demographics, surgical variables, postoperative outcomes, and postoperative follow-up. Specifically, we collected the following clinical, pathological, and surgical data: sex, age at the time of operation, body mass index (BMI), American Society of Anesthesiologists (ASA) classification of comorbidities [29], preoperative laboratory data (hemoglobin A1c after overnight fasting, carbohydrate antigen 19-9 (CA 19-9), carcinoembryonic antigen (CEA), albumin), neoadjuvant and adjuvant chemotherapy, operation type, length of hospital stay, tumor size and tumor, node, and metastasis (TNM) stages (American Joint Committee on Cancer, 8th edition) [30], tumor differentiation, lymphovascular invasion, perineural invasion, resection margin status, recurrence, and overall survival. With respect to the postoperative follow-up data, we measured the duration of survival after surgery from the time of surgery until death or the last visit to the outpatient department. The study was approved by the Institutional Review Board of Asan Medical Center, Seoul, South Korea (approval number: 2018-0901).

### 2.2. Statistical Analysis

We compared the demographic and perioperative data of the metformin group with those of a group receiving other medications. Continuous variables were analyzed using Student’s *t*-test and were presented as means ± standard deviation. Categorical variables were analyzed using the χ^2^ test and were presented as percentages. Differences with *p* values ≤ 0.05 were considered to be significant.

In the first model, we defined the metformin group (“ever”) as comprising those patients who received metformin at least once during the postoperative period and the other medication group (“never”) as patients who did not receive metformin postoperatively. The start and stop dates of medication were, however, not taken into consideration. On the basis of this “ever versus never” model, we prepared Kaplan–Meier curves for the recurrence-free survival and overall survival of these patients.

In the second model, we performed a time-varying analysis, while taking into account the start date of metformin use, but not the stop date. If patients in the metformin group discontinued the use of this drug or switched to using an alternative medication, they were placed in the other medication group. We performed a sensitivity analysis using the Cox proportional hazards model and extended the Cox model with time-varying covariates to compare recurrence-free and overall survivals based on metformin treatment. Some patients were initially classified as using metformin and others were classified as not using it. Patients who used metformin during the follow-up were switched to the metformin group at that time and remained as such until recurrence or death.

**Figure 1:**
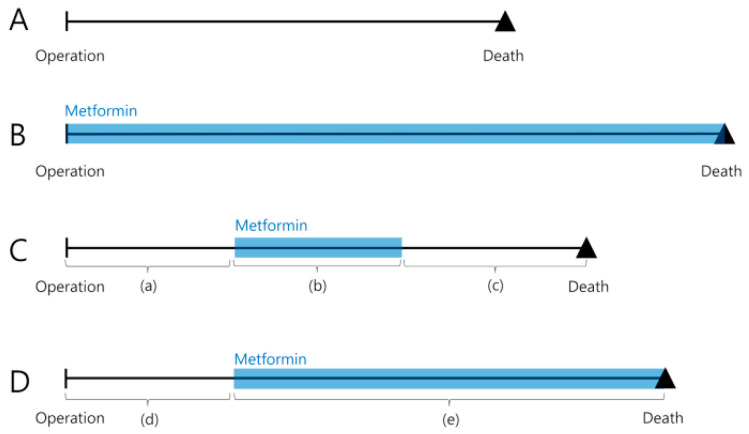
Illustration of the differences in metformin administration periods. (**A**) Patients who did not take metformin until death; (**B**) Patients who continuously took metformin between operation until death; (**C**) Patients who started taking metformin some time after operation and stopped before death; (**D**) Patients who started taking metformin some time after operation and continued until death.

In the third model, we performed a time-varying analysis, while taking into account both the start date and stop date of metformin use (Figure 1). Similar to the design of a previous study [31], the metformin group patients were placed in the other medication group upon discontinuing their use of metformin or switching to an alternative drug. The major difference between the second and the third models is the interval (c) shown in Figure 1 that considered the duration after the stop date of metformin (second model). The interval from (b) to (c) represents the metformin administration period as a whole, whereas the intervals (b) and (c) are distinguished from each other in the third model. The Cox model with time-varying covariates was used to compare the clinical outcomes based on metformin treatment.

Univariate and multivariate analyses were performed, and covariates were included in the multivariate analysis by using the backward elimination method. Adjusted analyses using Cox models with time-varying covariates are presented. After performing multivariate analysis, the interaction effects of metformin and clinical stage were analyzed to determine whether the clinical stages affected the relationship between metformin and the survival outcomes. Furthermore, we conducted a subgroup analysis to evaluate the dose-dependent effect of metformin. All statistical analyses were carried out in IBM SPSS version 24.0 (IBM Corp., Armonk, NY, USA) and SAS version 9.4 (SAS Institute, Cary, NC, USA).

## 3. Results

### 3.1. Patient Demographics

**Figure 2:**
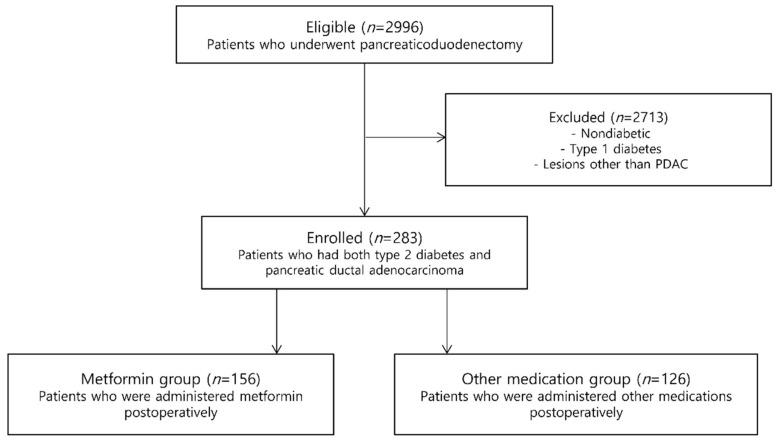
Flow chart summarizing the study identification and selection.

During the study period, a total of 2996 consecutive patients underwent PD, among whom 283 patients had both type 2 diabetes and PDAC. Of these patients, 157 (55.5%) had a history of postoperative metformin use, whereas the remaining 126 (44.5%) did not receive metformin postoperatively (Figure 2). Table 1 shows the baseline demographic and preoperative data of the metformin group and the other medication group. With the exception of ASA grade (*p* = 0.047), the two groups showed no significant differences in terms of sex, age at the time of operation, BMI, preoperative CA 19-9, CEA, albumin, or neoadjuvant chemotherapy.

Table 2 shows the postoperative data of the two medication groups. Although we detected no significant differences with respect to operation type, postoperative hospital stays, tumor size, TNM stage, differentiation, lymphovascular invasion, perineural invasion, or adjuvant chemotherapy, the R1 resection rate was significantly higher in the metformin group than that in the other medication group (31.2% vs. 20.6%, *p* = 0.045).

### 3.2. Survival Rate without Considering the Start and Stop Dates of Metformin Use (Ever Versus Never)

Initially, we compared the Kaplan–Meier curves of recurrence-free survival and overall survival between the two groups without taking into consideration the start and stop dates (Supplementary Figure S1), which revealed a significantly better survival in the metformin group. The median recurrence-free survival duration of the metformin group and the other medication group was 30.7 months (95% confidence interval (CI), 26.2–40.4 months) and 26.8 months (95% confidence interval (CI), 17.2–30.1 months), respectively; the 5-year recurrence-free survival rate of the metformin group and the other medication group was 19.4% and 11.0%, respectively; the median overall survival duration of patients in the metformin group and that of the patients in the other medication group was 63.7 months (95% confidence interval (CI), 52.7–82.4 months) and 45.5 months (95% confidence interval (CI), 35–56.6 months), respectively; and the 5-year overall survival rate of patients in the metformin group and the other medication group was 23.4% and 13.3%, respectively.

However, given that the Kaplan–Meier curves were constructed without considering the duration of medication, we subsequently performed further analyses using a Cox proportional hazards model and extended Cox model with time-varying covariates to adequately compare the recurrence-free survival and overall survival between the two groups.

### 3.3. Analysis Using Cox Models with Time-Varying Covariates Only Considering the Start Date of Metformin Use

Unadjusted analysis using Cox models with time-varying covariates, in which only the start date of postoperative metformin use was taken into consideration, revealed no significant differences with respect to either recurrence-free survival (Hazard ratio (HR), 0.903; 95% confidence interval (CI), 0.688–1.185; *p* = 0.463) or overall survival (HR, 0.862; 95% CI, 0.659–1.128; *p* = 0.280) between the two medication groups (Supplementary Table S1A).

Similarly, after univariate and multivariate analyses, adjusted analysis using Cox models with time-varying covariates considering only the start date of postoperative metformin use showed no significant difference in either recurrence-free survival (HR, 0.923; 95% CI, 0.691–1.234; *p* = 0.590) or overall survival (HR, 0.83; 95% CI, 0.627–1.098; *p* = 0.192) between the two groups (Table 3).

Analysis of the interaction between TNM stages and the effect of metformin on clinical outcomes revealed no significant interactions (*p* = 0.103 for recurrence-free survival, *p* = 0.170 for overall survival) (Supplementary Table S6), nor were we able to detect any significant trends in the interaction effect (*p* = 0.208 for recurrence-free survival, *p* = 0.262 for overall survival) (Supplementary Table S6).

### 3.4. Analysis Using Cox Models with Time-Varying Covariates Considering Both the Start Date and Stop Date of Metformin Use

Unadjusted analysis using Cox models with time-varying covariates, in which both the start date and stop date of postoperative metformin use were taken into consideration, indicated no significant difference in recurrence-free survival (HR, 0.955; 95% CI, 0.731–1.249; *p* = 0.737) between the two medication groups. However, the same analysis with respect to overall survival revealed a significant difference between the two groups (HR, 0.712; 95% CI, 0.543–0.933; *p* = 0.014) (Supplementary Table S1B).

Given that we detected significant differences in ASA and resection margin status between the two groups (Table 1 and Table 2) and that BMI and preoperative hemoglobin A1c are crucial factors for predicting clinical outcomes, we included these variables in the univariate and multivariate analyses of recurrence-free survival and overall survival (Supplementary Tables S2–S5). The results obtained from the multivariate analysis indicated that ASA, BMI, preoperative hemoglobin A1c, preoperative metformin use, and resection margin status had no significant effects on either recurrence-free survival or overall survival. In contrast, although adjusted analysis using Cox models with time-varying covariates considering both the start date and stop date of postoperative metformin revealed no significant difference in recurrence-free survival (HR, 1.047; 95% CI, 0.783–1.4; *p* = 0.754) between the two medication groups, it did indicate a significant difference in overall survival (HR, 0.747; 95% CI, 0.562–0.993; *p* = 0.045) (Table 3).

We detected no significant interactions among the TNM stages with respect to survival (*p* = 0.593 for recurrence-free survival, *p* = 0.060 for overall survival) (Supplementary Table S7), nor were there any significant trends in the interaction effects (*p* = 0.299 for recurrence-free survival, *p* = 0.273 for overall survival) (Supplementary Table S7). Furthermore, subgroup analysis of the dose-dependent effect of metformin revealed no significant difference in the recurrence-free survival or overall survival with respect to metformin dosage (Supplementary Table S8).

## 4. Discussion

Subsequent to the study by Evans et al., in which the authors reported that metformin causes a significant reduction in the incidence of cancer [32], numerous retrospective studies, meta-analyses, and reviews have been performed to assess the beneficial effects of metformin on the prevention and treatment of pancreatic cancer [11,12,13,14,18,19,20,21,22,23,24]. However, three RCTs on combination therapy using metformin in conjunction with chemotherapeutic agents for advanced pancreatic cancer patients failed to show any significant beneficial effect on clinical outcomes [15,17,26], whereas Suissa et al. warned that time-related bias can undermine the results of observational studies on metformin [25]. Moreover, Chaiteerakij et al. have highlighted the problem of dichotomous categorization (ever versus never) of metformin use in conventional Cox proportional hazards regression models and claimed that there is no significant survival benefit of metformin in patients with pancreatic cancer [27].

Nevertheless, numerous in vivo and in vitro studies have demonstrated the cytotoxic effect of metformin on pancreatic cancer cells [1,2,3,4,5,6,7]. In this respect, the results of previous laboratory studies have indicated that metformin exerts a concentration-dependent cytotoxic effect on pancreatic cancer cells, and promotes significant activation of the AMPK pathway and downregulation of mTOR signaling, an important downstream effector of the PI3K/Akt pathway [3,33,34,35,36]. Activated AMPK is associated with gluconeogenesis, lipogenesis, protein synthesis, and angiogenesis, and physiologically inhibits mTOR signaling, which is a critical pathway with respect to the prognosis of pancreatic cancer [36]. It should be noted, however, that in the three aforementioned RCTs that failed to show a beneficial effect of metformin, the patients were only treated using metformin in combination with other chemotherapeutic agents, and that in all these patients pancreatic cancer was at an advanced stage [15,17,26]. Moreover, while Chaiteerakij et al. pointed out an important limitation of previous retrospective studies on metformin, they only considered the starting date of metformin administration and not the stop date [27]. In clinical situations, even if two given patients are initiated on metformin on the same day, they should be dealt with differently if there is a significant difference with respect to the dates on which metformin administration is discontinued. Under these circumstances, RCTs that enroll patients with operable pancreatic cancer are needed to identify the actual effect of metformin as a postoperative therapy following pancreatic resection in pancreatic cancer patients. Thus, as a preliminary study for a future RCT, we conducted a retrospective cohort study using time-dependent analysis.

If patients follow progression A or B depicted in Figure 1, the dichotomous categorization of ever/never regarding metformin use may be appropriate. However, in clinical situations, patients may be characterized by sequences C or D, which warrants analysis based on Cox models with time-varying covariates, as previously suggested [25,27]. Using this approach, the guarantee-time bias may be overcome by considering metformin administration as a time-dependent covariate [37]. However, if we were only to consider the start date of metformin administration into consideration, we would be unable to differentiate patients characterized by courses C and D in Figure 1. To overcome this problem, we collected data regarding both the start and stop dates of metformin administration and defined a new time-varying covariate that incorporates both dates. Thus, all intervals ((a), (b), (c), (d), and (e)) in Figure 1 were dealt with as individual entities in the final analysis.

Similar to the results obtained by Chaiteerakij et al., we found that adjusted analysis using Cox models with time-varying covariates, in which only the start date of postoperative metformin use was considered, revealed no significant difference in recurrence-free survival and overall survival between the metformin and other medication groups (Table 3). However, analysis in which both the start date and stop date were taken into consideration indicated a significant difference in overall survival between the two groups (*p* = 0.045) (Table 3). Nevertheless, even though the positive resection margin rate was significantly higher in the metformin group (Table 2), we detected no significant effect of resection margin on either the recurrence-free survival or overall survival on the basis of multivariate analysis (Supplementary Tables S2–S5). However, the R1 resection rate was observed to be higher in the metformin group, and the overall survival rate was also found to be higher in this group, which could be considered as persuasive evidence of the anticancer effect of metformin in the treatment of pancreatic cancer. These results accordingly indicate the survival benefits obtained from metformin following PD in type 2 diabetic patients with PDAC. Moreover, it is conceivable that different results might have been obtained in previous studies had the authors incorporated both the start and stop dates of medication using time-varying analysis. However, as we did not observe significant benefits in recurrence-free survival, it remains unclear as to whether postoperative metformin use is effective for PDAC. It is plausible that metformin may not be sufficiently potent to reduce the recurrence after PD, but remains effective with respect to lengthening overall survival by reducing the tumor burden.

Results obtained in previous studies have tended to differ depending on the type of the analytical method used. Specifically, studies that performed analysis according to ever/never metformin treatment have shown the significant clinical benefit of metformin treatment [11,12,13,14,15,26,38,39,40,41,42], whereas those that have used Cox models with time-varying covariates considering the start date of metformin use have found no significant differences based on metformin treatment [24,27,43,44]. Although the more simplistic analysis of ever/never metformin use may be more prone to bias, each of the two analytical methods has its own strengths, and thus it is difficult to conclude that one method is better than the other. Based on the comprehensive review of studies on metformin and PDAC, we established that our study is the first that has performed analysis using Cox models with time-varying covariates that incorporate both the start and stop dates of metformin use. Furthermore, whereas previous studies have analyzed the effects of metformin without any regard to the timing of administration, we specifically included patients who only received metformin following surgery to investigate the effect of postoperative metformin use on oncological outcomes. To gain further evidence on the real-world effect of metformin following PD in type 2 diabetic patients with PDAC, it will be necessary to conduct a larger number of retrospective studies incorporating both the treatment start and stop dates to provide a more definitive conclusion.

The present study does, however, have certain limitations. In addition to metformin, patients also received the following medications for glucose control: sulfonylureas (e.g., glimepiride), meglitinides (e.g., nateglinide, repaglinide), thiazolidinediones (e.g., lobeglitazone and pioglitazone), α-glucosidase inhibitors (e.g., acarbose), dipeptidyl peptidase IV inhibitors (e.g., anagliptin and vildagliptin), and insulin. It is conceivable that these medications could have modified the effect of metformin and thereby influenced the clinical outcomes. Furthermore, even though we used time-varying covariate analysis, this study still has limitations inherent in its retrospective design based on a cohort from a single center. To overcome these limitations—as much as possible—we conducted comprehensive research on the administration period of metformin by reviewing electronic medical records and conducting telephone inquiries. Regarding our analysis using Cox models with time-varying covariates considering both the start and stop dates of metformin, it may be debatable as to whether intervals (a) and (c) in Figure 1 can be independent. Given that our patients received metformin during interval (b), the clinical outcomes of the patients during interval (c) may be affected by this. However, the effect of the remaining metformin would be insignificant as the half-life of this drug is between 3 and 5.7 h [45,46,47] and metformin is generally not present in the plasma 24 h after oral administration [45].

In conclusion, our results indicate that metformin may be beneficial as a postoperative therapy for improving overall survival following PD in patients with type 2 diabetes and PDAC. Further studies using time-varying analysis considering both the start and stop dates of medication and well-designed RCTs for operable pancreatic cancer are needed in order to provide further evidence on the beneficial effects of metformin following PD in type 2 diabetes patients with PDAC.

## Figures and Tables

**Table 1:** Demographic and preoperative data of the metformin group and the other medication group.

Characteristics	Metformin Group (*n* = 157)	Other Medication Group (*n* = 126)	*p* Value
Sex, *n* (%)			0.272
Female	61 (38.9)	41 (32.5)	
Male	96 (61.1)	86 (67.5)	
Age, years ± SD	64.6 ± 8.3	63.9 ± 9.2	0.510
BMI, kg/m^2^ ± SD	22.4 ± 3.0	22.8 ± 2.9	0.242
ASA, *n* (%)			0.047
Grade I	0 (0)	0 (0)	
Grade II	145 (92.4)	107 (84.9)	
Grade ≥ III	12 (7.6)	19 (15.1)	
Preoperative HbA1c	8.3 ± 1.6	8.1 ± 1.6	0.578
Preoperative CA19-9, U/mL	566.7 ± 1481.1	1016.5 ± 2943.5	0.123
Preoperative CEA, ng/mL	10.6 ± 71.9	4.4 ± 3.7	0.358
Preoperative Albumin, g/dL	3.4 ± 0.5	3.3 ± 0.5	0.101
Neoadjuvant chemotherapy, *n* (%)			0.462
(+)	9 (5.7)	10 (7.9)	
(−)	148 (94.3)	116 (92.1)	

SD, standard deviation; HbA1c, hemoglobin A1c; CA19-9, carbohydrate antigen 19-9; CEA, carcinoembryonic antigen.

**Table 2:** Postoperative data of the metformin group and the other medication group.

Characteristics	Metformin Group (*n* = 157)	Other Medication Group (n = 126)	*p* Value
Operation type, *n* (%)			0.496
Laparoscopy	3 (1.9)	4 (3.2)	
Open	154 (98.1)	122 (96.8)	
Postoperative hospital stay, *n* ± SD	16.1 ± 8.3	17.2 ± 10.3	0.327
Tumor size, cm ± SD	3.2 ± 1.0	3.3 ± 1.1	0.822
TNM stage, *n* (%)			0.924
IA	10 (6.4)	5 (4.0)	
IB	43 (27.4)	33 (26.2)	
IIA	7 (4.5)	4 (3.2)	
IIB	66 (42.0)	56 (44.4)	
III	31 (19.1)	27 (21.4)	
IV	1 (0.6)	1 (0.8)	
Differentiation, *n* (%)			0.734
Well	17 (11.4)	14 (11.6)	
Moderate	112 (75.2)	88 (72.7)	
Poor	19 (12.8)	19 (15.7)	
Undifferentiated	1 (0.7)	0 (0)	
Lymphovascular invasion, *n* (%)			0.258
(+)	94 (59.9)	67 (53.2)	
(−)	63 (40.1)	59 (46.8)	
Perineural invasion, *n* (%)			0.640
(+)	133 (84.7)	109 (86.5)	
(−)	24 (15.3)	17 (13.5)	
Resection margin status, *n* (%)			0.045
(+)	49 (31.2)	26 (20.6)	
(−)	108 (68.8)	100 (79.4)	
Adjuvant chemotherapy, *n* (%)			0.464
(+)	105 (66.9)	79 (62.7)	
(−)	52 (33.1)	46 (37.3)	
Chemotherapy before recurrence, *n* (%)			0.253
(+)	83 (52.9)	58 (46.0)	
(−)	74 (47.1)	68 (54.0)	

SD, standard deviation; TNM, tumor, node, metastasis.

**Table 3:** Adjusted analysis using Cox models with time-varying covariates.

Outcome	Metformin Use	Hazard Ratio (95% Confidence Interval)	*p* Value
Only considering the start date of metformin use			
Recurrence-free survival	(+)	0.923 (0.691–1.234)	0.590
	(−)	1	
Overall survival	(+)	0.83 (0.627–1.098)	0.192
	(−)	1	
Considering both the start date and stop date of metformin use			
Recurrence-free survival	(+)	1.047 (0.783–1.400)	0.754
	(−)	1	
Overall survival	(+)	0.747 (0.562–0.993)	0.045
	(−)	1	

## Supplementary Materials

The following are available online at https://www.mdpi.com/2077-0383/9/6/1953/s1, Table S1: Unadjusted analysis using Cox models with time-varying covariates, Table S2: Univariate analysis of recurrence-free survival, Table S3: Multivariate analysis of recurrence-free survival with covariates selected using the backward elimination method, Table S4: Univariate analysis of overall survival, Table S5: Multivariate analysis of overall survival with covariates selected using the backward elimination method, Table S6: Analysis of the interactive effect of clinical stages on clinical outcomes considering the start date of metformin use, Table S7: Analysis of the interactive effect of clinical stages on clinical outcomes considering both the start and stop dates of metformin use, Table S8: Subgroup analysis of the dose-dependent effect of metformin, Figure S1: (**A**) Kaplan-Meier curves of recurrence-free survival after surgery in the metformin group and the other medication group. (**B**) Kaplan-Meier curves of overall survival after surgery in the metformin group and the other medication group.
