# Supplementary material for: Association between Metformin Use and Clinical Outcomes Following Pancreaticoduodenectomy in Patients with Type 2 Diabetes and Pancreatic Ductal Adenocarcinoma
Source: J Clin Med. 2020 Jun 22;9(6):1953. doi: 10.3390/jcm9061953 (PMC7356590; doi:10.3390/jcm9061953)
Supplement: Supplementary file 1 [file jcm-09-01953-s001.pdf]

Appendix

Supplementary Figure 1. (A) Kaplan-Meier curves of recurrence-free survival after surgery in the metformin group and the other medication group. (B) Kaplan-Meier curves of overall survival after surgery in the metformin group and the other medication group.

(A)

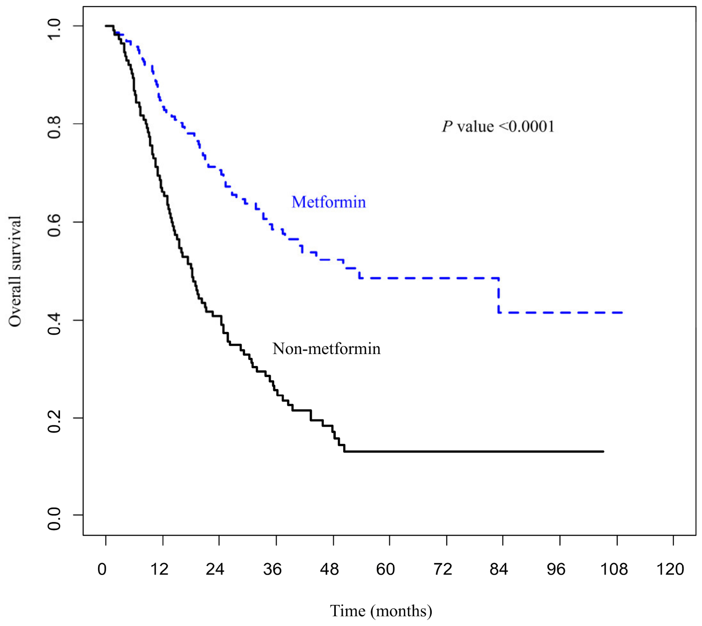

(B)

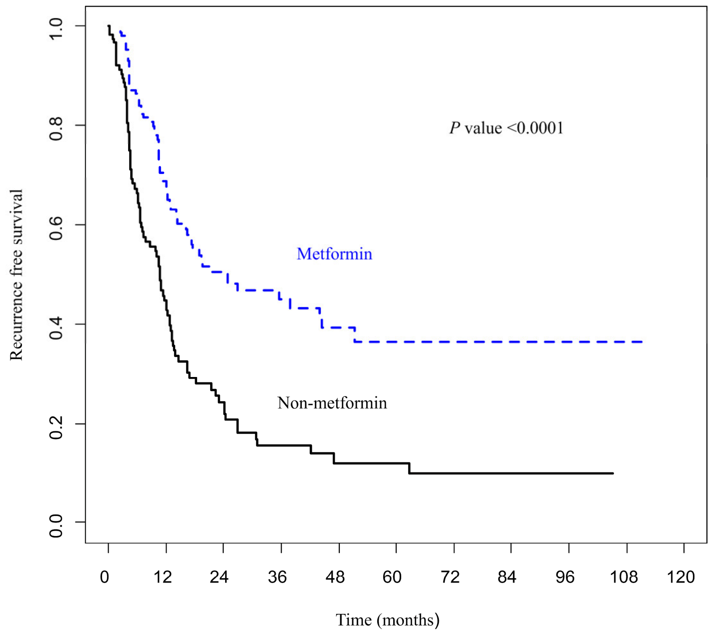

Supplementary Table 1. Unadjusted analysis using Cox models with time-varying covariates

(A) Only considering the start date of metformin use

**Outcome**	**Metformin use**	**Hazard ratio (95% confidence interval)**	***P* value**
Recurrence-free survival	(+)	0.903 (0.688–1.185)	0.463
	(-)	1	
Overall survival	(+)	0.862 (0.659–1.128)	0.280
	(-)	1	

(B) Considering both the start date and stop date of metformin use

**Outcome**	**Metformin use**	**Hazard ratio (95% confidence interval)**	***P* value**
Recurrence-free survival	(+)	0.955 (0.731–1.249)	0.737
	(-)	1	
Overall survival	(+)	0.712 (0.543–0.933)	0.014
	(-)	1	

Supplementary Table 2. Univariate analysis of recurrence-free survival

Variables		Hazard ratio (95% confidence interval)	*P* value
Metformin use	(+)	0.799 (0.605–1.057)	0.116
	(-)	1	
Sex	Female	1.077 (0.81–1.432)	0.611
	Male	1	
Age		0.995 (0.978–1.012)	0.573
BMI		0.992 (0.945–1.041)	0.739
ASA	Grade II	1	
	Grade ≥III	0.714 (0.457–1.115)	0.139
Preoperative HbA1c		1.089 (0.988 – 1.201)	0.085
Preoperative CA19-9		1	0.069
	Per 10 units	1.000 (1.000–1.001)	
	Per 100 units	1.004 (1.000–1.009)	
Preoperative albumin		0.904 (0.689–1.188)	0.470
Neoadjuvant chemotherapy	(+)	0.974 (0.555–1.709)	0.927
	(-)	1	
Preoperative metformin use	(+)	0.852 (0.646–1.124)	0.257
	(-)	1	
Operation type	Laparoscopy	0.638 (0.252–1.550)	0.321
	Open	1	
Tumor size		1.289 (1.136–1.462)	< 0.0001
TNM stage	I	1	< 0.0001
	II	1.984 (1.422–2.766)	< 0.0001
	III	3.192 (2.120–4.806)	< 0.0001
	IV	2.673 (0.649–11.001)	< 0.173
Differentiation	Well	1	< 0.0001
	Moderate	1.404 (0.895–2.204)	0.140
	Poor	3.103 (1.798–5.355)	< 0.0001
Lymphovascular invasion	(+)	1.449 (1.094–1.919)	0.010
	(-)	1	
Perineural invasion	(+)	2.097 (1.333–3.299)	0.010
	(-)	1	
Resection margin status	(+)	1.414 (1.039–1.925)	0.028
	(-)	1	

NA, not applicable

Supplementary Table 3. Multivariate analysis of recurrence-free survival with covariates selected using the backward elimination method

**Variables**		**Hazard ratio (95% confidence interval)**	***P* value**
Metformin use	(+)	0.865 (0.642–1.167)	0.343
	(-)	1	
Sex	Female		
	Male		
Age			
BMI			
ASA	Grade II		
	Grade ≥III		
Preoperative HbA1c			
Preoperative CA19-9	Per 10 units		
	Per 100 units		
Preoperative albumin			
Neoadjuvant chemotherapy	(+)		
	(-)		
Preoperative metformin use	(+)		
	(-)		
Operation type	Laparoscopy		
	Open		
Tumor size		1.284 (1.103–1.494)	0.001
TNM stage	I	1	< 0.0001
	II	1.683 (1.179–2.403)	0.004
	III	2.915 (1.91–4.447)	< 0.0001
	IV	4.656 (1.046–20.724)	0.044
Differentiation	Well	1	< 0.0001
	Moderate	1.383 (0.872–2.193)	0.168
	Poor	3.542 (2.028–6.188)	< 0.0001
Lymphovascular invasion	(+)		
	(-)		
Perineural invasion	(+)	1.875 (1.116–3.149)	0.018
	(-)	1	
Resection margin status	(+)		
	(-)		

NA, not applicable

Supplementary Table 4. Univariate analysis of overall survival

Variables		Hazard ratio (95% confidence interval)	*P* value
Metformin use	(+)	0.712 (0.543–0.933)	0.014
	(-)	1	
Sex	Female	1.077 (0.818–1.419)	0.597
	Male	1	
Age		1.011 (0.994–1.029)	0.193
BMI		0.995 (0.949–1.044)	0.842
ASA	Grade II	1	
	Grade ≥III	0.645 (0.427–0.975)	0.038
Preoperative HbA1c		1.066 (0.971 – 1.171)	0.180
		1	0.309
Preoperative CA19-9	Per 10 units	1.000 (1.000–1.001)	
	Per 100 units	1.003 (0.998–1.008)	
Preoperative albumin		0.691 (0.527–0.905)	0.007
Neoadjuvant chemotherapy	(+)	1.127 (0.667–1.906)	0.654
	(-)	1	
Preoperative metformin use	(+)	0.854 (0.653–1.116)	0.247
	(-)	1	
Operation type	Laparoscopy	0.526 (0.195–1.417)	0.204
	Open	1	
Tumor size		1.306 (1.167–1.460)	< 0.0001
TNM stage	I	1	< 0.0001
	II	1.717 (1.241–2.375)	0.001
	III	3.339 (2.281–4.888)	< 0.0001
	IV	0.96 (0.133–6.944)	0.968
Differentiation	Well	1	< 0.0001
	Moderate	1.358 (0.868–2.126)	0.180
	Poor	2.566 (1.497–4.398)	0.001
Lymphovascular invasion	(+)	1.123 (0.858–1.47)	0.397
	(-)	1	
Perineural invasion	(+)	1.481 (0.983–2.234)	0.061
	(-)	1	
Resection margin status	(+)	1.535 (1.146–2.057)	0.004
	(-)	1	

NA, not applicable

Supplementary Table 5. Multivariate analysis of overall survival with covariates selected using the backward elimination method

**Variables**		**Hazard ratio (95% confidence interval)**	***P* value**
Metformin use	(+)	0.747 (0.562–0.993)	0.045
	(-)	1	
Sex	Female		
	Male		
Age		1.038 (1.018–1.058)	0.001
BMI			
ASA	Grade II		
	Grade ≥III		
Preoperative HbA1c			
Preoperative CA19-9	Per 10 units		
	Per 100 units		
Preoperative albumin		0.745 (0.549–1.011)	0.058
Neoadjuvant chemotherapy	(+)	1.748 (1.019–3.000)	0.043
	(-)	1	
Preoperative metformin use	(+)		
	(-)		
Operation type	Laparoscopy		
	Open		
Tumor size		1.33 (1.165–1.518)	< 0.0001
TNM stage	I	1	< 0.0001
	II	1.529 (1.071–2.184)	< 0.0001
	III	3.877 (2.527–5.947)	0.874
	IV	0.852 (0.117–6.203)	0.024
Differentiation	Well	1	< 0.0001
	Moderate	1.685 (1.070–2.653)	0.024
	Poor	3.539 (2.022–6.194)	< 0.0001
Lymphovascular invasion	(+)		
	(-)		
Perineural invasion	(+)		
	(-)		
Resection margin status	(+)		
	(-)		

NA, not applicable

Supplementary Table 6. Analysis of the interactive effect of clinical stages on clinical outcomes considering the start date of metformin use

**Stage**	**Recurrence-free survival**		**Overall survival**	
	**Hazard ratio**	***P* value**	**Hazard ratio**	***P* value**
Stage 1	0.582 (0.338–1.003)	0.051	0.572 (0.337–0.973)	0.039
Stage 2	1.214 (0.804–1.834)	0.356	1.085 (0.723–1.627)	0.695
Stage 3 or 4	0.805 (0.432–1.501)	0.495	0.787 (0.446–1.336)	0.406
Interaction *P* value (Stage 1/2/3 or 4)		0.103		0.170
Interaction *P* value (Trend)		0.208		0.262

Supplementary Table 7. Analysis of the interactive effect of clinical stages on clinical outcomes considering both the start and stop dates of metformin use

**Stage**	**Recurrence-free survival**		**Overall survival**	
	**Hazard ratio**	***P* value**	**Hazard ratio**	***P* value**
Stage 1	0.819 (0.471–1.424)	0.480	0.452 (0.254–0.806)	0.007
Stage 2	1.162 (0.774–1.745)	0.469	1.041 (0.696–1.556)	0.847
Stage 3 or 4	1.115 (0.598–2.077)	0.733	0.668 (0.370–1.204)	0.180
Interaction *P* value (Stage 1/2/3 or 4)		0.593		0.060
Interaction *P* value (Trend)		0.299		0.273

Supplementary Table 8. Subgroup analysis of the dose-dependent effect of metformin

Outcome		Mean dose	Standard deviation	Hazard ratio	Hazard ratio 95% confidence interval	*P* value
Overall survival	1	984.0	500.6	1.01	0.98–1.05	0.51
	0	1004.6	501.9			
Recurrence-free survival	1	1009.9	498.5	1.01	0.97–1.04	0.71
	0	994.7	502.7			
